# An Enhanced Method to Evaluate Tensile Yield Stress by Small Punch Tests Using Deflection Curves

**DOI:** 10.3390/ma13122840

**Published:** 2020-06-24

**Authors:** Betül Gülçimen Çakan, Peter Hähner, Celal Soyarslan, Swantje Bargmann

**Affiliations:** 1 Department of Mechanical Engineering, Bursa Uludağ University, Görükle, 16059 Bursa, Turkey; bgulcimen@uludag.edu.tr; 2 Nuclear Safety and Security Directorate, Joint Research Centre, European Commission, NL-1755 LE Petten, The Netherlands; 3 School of Mechanical Engineering and Safety Engineering, University of Wuppertal, 42119 Wuppertal, Germany; soyarslan@uni-wuppertal.de (C.S.); bargmann@uni-wuppertal.de (S.B.)

**Keywords:** small punch test, deflection, yield stress, finite element method

## Abstract

While force–displacement curves are often preferred in Small Punch (SP) tests due to the ease of the experimental set-up, they encompass significant uncertainties arising from frame compliance. In this work, a methodology is presented to predict yield stresses from the force vs. deflection curves. The present method relies on determining different force levels from the initial part of the force–deflection curve to reflect both the slope and the curvature instead of using a single force level only. The predicted yield stresses for different types of materials, that is, low- and high-strength alloys, are found to be in good agreement with the actual proof stresses with a maximum error of 16%.

## 1. Introduction

Structural integrity assessment is critical for ensuring the safe operation and determining the residual lifetime of, for instance, power plant components. Various standard tests, for example, tensile and Charpy impact tests, can be performed to obtain mechanical properties. Still, material sampling for the standard tests would require material volumes that are too large to be extracted from in-service components. Thus miniaturized testing techniques have been developed to enable sample extraction of limited quantities. These techniques either employ small-sized specimens of the conventional form (e.g., micro tensile, sub-sized Charpy, or compact tension tests) or innovative specimen designs [1].

Small Punch (SP) testing using disc-shaped specimens with a thickness of 0.25 or 0.5 mm and diameters of 3 or 8 mm, respectively, is an example of the latter approach. Due to the small size of the specimens, this technique enables extracting samples from local areas like a weld or even the heat-affected zone of a weldment [2,3,4], where the non-uniformity of the mechanical properties precludes usage of standard specimen sizes. SP testing has also proven important a technique for rapid prototyping and virtually non-destructive performance assessment of service-exposed materials. With the adoption of the draft European standard CEN—prEN 10371 on Metallic materials-Small punch test method, SP testing is expected to further gain importance also for design purposes, provided that robust methods are available for the correlation of SP test results with conventional tensile test results. In this sense, the present work is to contribute a robust method for the derivation of tensile yield stresses from SP tests.

In an SP fracture test, one applies a constant displacement rate to a hemispherical-punch acting on the center of a rigidly clamped SP disc until the specimen fails. During testing, one records force–displacement and/or force–deflection data, with deflection being measured as the movement of the apex of the disc dome as it bulges out. In order to develop a relationship, both tensile and SP tests are carried out for a particular material, and tensile properties, in particular, yield stress ($\sigma_{y}$) and ultimate tensile strength are correlated with characteristic forces and dimensions featured by the SP fracture test data, that is, the elastic–plastic transition force ($F_{e}$) and the maximum force, respectively, using empirical or semi-empirical relations [2,5,6,7,8,9,10,11,12,13,14]. There are also studies where the conventional SP sample geometry has been modified for the correlation with fracture tests [15,16].

For the yield stress correlation, researchers have mostly used the relationship proposed by Mao [5]

(1) $$\sigma_{y}=\alpha \frac{F_{e}}{h^{2}}\;.$$

Here *h* is the initial disc thickness and α a dimensionless material coefficient. In the authors’ previous work [17], it was shown that this coefficient should not be taken as a constant, and instead, a wide range of α values has been determined experimentally for various materials. A new methodological scheme to predict the yield stress from SP curves has then been developed. Instead of using a single force level, this scheme used a triplet of forces (curvature factor) reflecting the curvature of the force–displacement curve.

In the present study, the methodology is further enhanced by extending it to the use of force–deflection curves instead of force–displacement curves. The aim of using deflection curves was to improve the prediction accuracy by eliminating the initial plastic indentation (as visible experimentally and numerically in finite element models) and frame compliance effects (characteristic of real experiments). The new scheme comprises both a curvature- and a slope-dependent coefficient, which best reflect the characteristics of low- and high-strength metals, respectively.

## 2. Comparison of Displacement- vs. Deflection-Based Approaches

**Figure 1:**
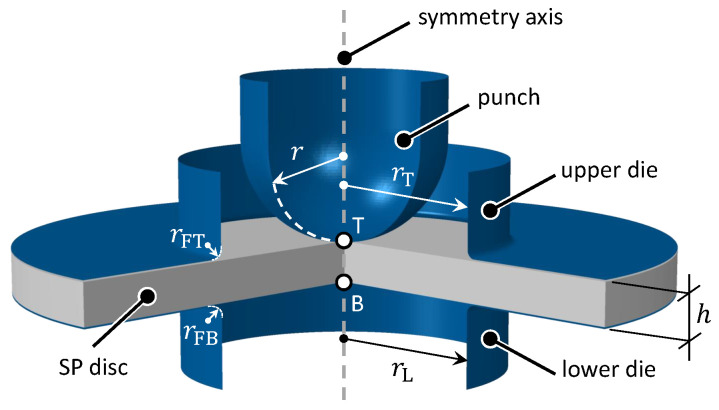
A schematic depiction of the Small Punch (SP) test for which an axisymmetric finite element model is developed. Here, the punch and the dies are modelled as rigid surfaces. The SP disc is modelled as a deformable body which is uniformly discretised using CAX4R elements with element size of 0.005 mm. At the central point at the top surface, denoted by T, *displacement* readings for an ideally stiff loading frame are realized whereas *deflection* readings are realized at the central point at the bottom surface, denoted by B.

In practice, either displacement or deflection of the SP specimen can be measured to quantify the deformation. In this work, we use the notation introduced in Figure 1 in which the SP test is depicted. Accordingly, the displacement corresponds to the vertical translation of the center at the top face of the specimen denoted by T. In contrast, deflection is the vertical translation of the central point at the opposite, that is, the bottom face, denoted by B. Displacement can be measured by a displacement transducer, for example, linear variable displacement transducer (LVDT), mounted close to the specimen or by recording the movement of the machine cross-head. Depending on the position of the displacement transducer and the stiffness of the loading frame, the observed displacement values need to be corrected significantly to account for the frame compliance including any compliance of the punch itself.

In the case of deflection, the translation of the bottom of the specimen can directly be recorded by an LVDT or an extensometer that is placed underneath the specimen. As an advantage, this configuration does not require any parasitic compliance correction, and hence reflects the actual material response. As a disadvantage, this amounts to a more complicated test set-up, in particular, for high-temperature test applications. Because of its more straightforward implementation, most researchers have preferred measuring displacement [2,8,9,10]. Nevertheless, considering the small range of deformation in the SP test, in particular, at the initial stage of relevance to yielding, the accuracy of the displacement measurement in practice is strongly affected by the frame compliance, thereby limiting the precision to which the yield stress can be determined.

Some researchers have remarked on the compliance effect [11,18]. Campitelli et al. showed that load train compliance effects are not negligible, and measuring the deflection from the bottom is recommended in particular for the first part of the deformation curve [18]. Moreno [14] also emphasized that the readings should be done from the bottom of the specimen so that the plastic indentation will not be included, and it will purely reflect the elastic regime. In the work in References [12,19], the authors used an experimental set-up with LVDTs to monitor both displacement and deflection, which enabled them to monitor the thinning of the disc which accompanies the plastic deformation. Janca et al. [12] developed a methodology to the predict yield stress from force vs. thinning curves instead of standard force–displacement curves. By contrast, Chica et al. [13] stated that both displacement and deflection curves resulted in the same accuracy of yield stress estimation and favoured displacement measurement for its simplicity.

## 3. Numerical Model

Similar to the authors’ previous works [17,20,21], models are developed using a 2D axisymmetric idealization. As demonstrated in Figure 1, the configuration consists of a deformable disc with a thickness of h=0.5 mm, which is rigidly clamped by upper and lower dies having an aperture of $2r_{T}=2r_{B}=4$ mm. Fillets of $r_{\mathrm{FT}}=r_{\mathrm{FB}}=0.1$ mm are applied. The hemispherical punch of r=1.25 mm of radius penetrates the disc at a constant velocity of 0.005 mm/s. The friction coefficient between the punch and the disc was set to 0.25, noting that friction effects are insignificant at the low force levels of interest for yielding. As for the die-disc interface the friction coefficient was set to 1 in order to ensure the slip-free clamping in the numerical experiments. A uniform mesh with element size 0.005 mm and element-type CAX4R, that is, 4-node, reduced-integration, first-order, axisymmetric solid element, was sed.

Here, the punch and the dies are modelled as rigid surfaces. The SP disc is modelled as a deformable body which is uniformly discretised using CAX4R elements with element size of 0.005 mm. At the central point at the top surface, denoted by T, *displacement* readings are realized whereas *deflection* readings are realized at the central point at the bottom surface, denoted by B.

The hardening behaviour of the material was introduced as power-law hardening also known as Hollomon rule [22]

(2) $$\sigma =C\epsilon^{n}\;,$$

where σ is the Cauchy stress and ϵ is the plastic strain with strength parameters and hardening exponents, *C* and *n* in the range from 300 to 1500 MPa, and 0.02 to 0.6, respectively. With E=70 GPa, 210 GPa and 400 GPa, three different levels of elastic moduli *E* have been chosen. This choice of material parameters is representative of the entire actual range of metallic materials from annealed low-strength metals to tempered high strength alloys. Matlab scripts were developed to automatize the simulation runs with different parameters and identify different force levels from numerical force–deflection curves.

## 4. Results

### 4.1. Analysis of Deflection Data with the Displacement-Based Formula

Force-displacement curves are popular due to the easy experimental set-up. However, they are sensitively affected by frame compliance effects, the accurate correction of which is difficult in practice. As a disadvantage, large errors can be introduced in the analysis of results.

Force–deflection curves do not suffer from this disadvantage. Here they were generated numerically for the mentioned ranges of *C*, *n*, and *E*. Initially, the curves were evaluated according to the previously developed formulas based on ideal force–displacement curves for an infinitely stiff frame [17]. Henceforth, we refer to this formulation to predict the yield stress ($\sigma_{y}$) as the *displacement-based formula*.

(3) $$\sigma_{y}=\left\{\begin{array}{cc} \left[1.28K-0.062\right]\frac{F_{50}}{h^{2}} & \mathrm{for}\;K<0.33\;,\\ 0.360\frac{F_{50}}{h^{2}} & \mathrm{otherwise}\;. \end{array}\right.$$

**Figure 2:**
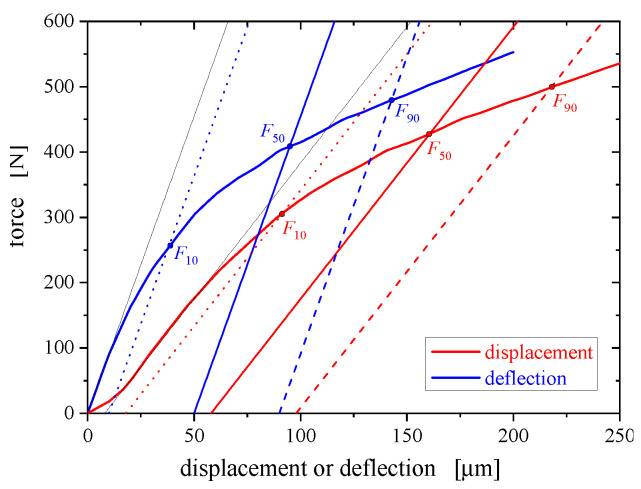
Force levels at 10, 50, 90 μm offsets applied to force vs. displacement and deflection curves, respectively: (i) The initial settling due to imperfections in load transfer across interfaces leads to a positive initial curvature, and subsequently an inflection point. (ii) The frame compliance (if not corrected properly) leads to a smaller slope at the inflection point, as compared to the initial slope vs. deflection.

Herein, the curvature factor *K* is identified using a triplet of force levels at displacement offsets of 10, 50 and 90 μm. For the definition of these forces the reader is referred to Figure 2, which also illustrates apparent differences between displacement and deflection.

(4) $$K=2\frac{F_{50}-F_{10}}{F_{90}-F_{10}}-1=\frac{\left[F_{50}-F_{10}\right]-\left[F_{90}-F_{50}\right]}{F_{90}-F_{10}}\;.$$

**Figure 3:**
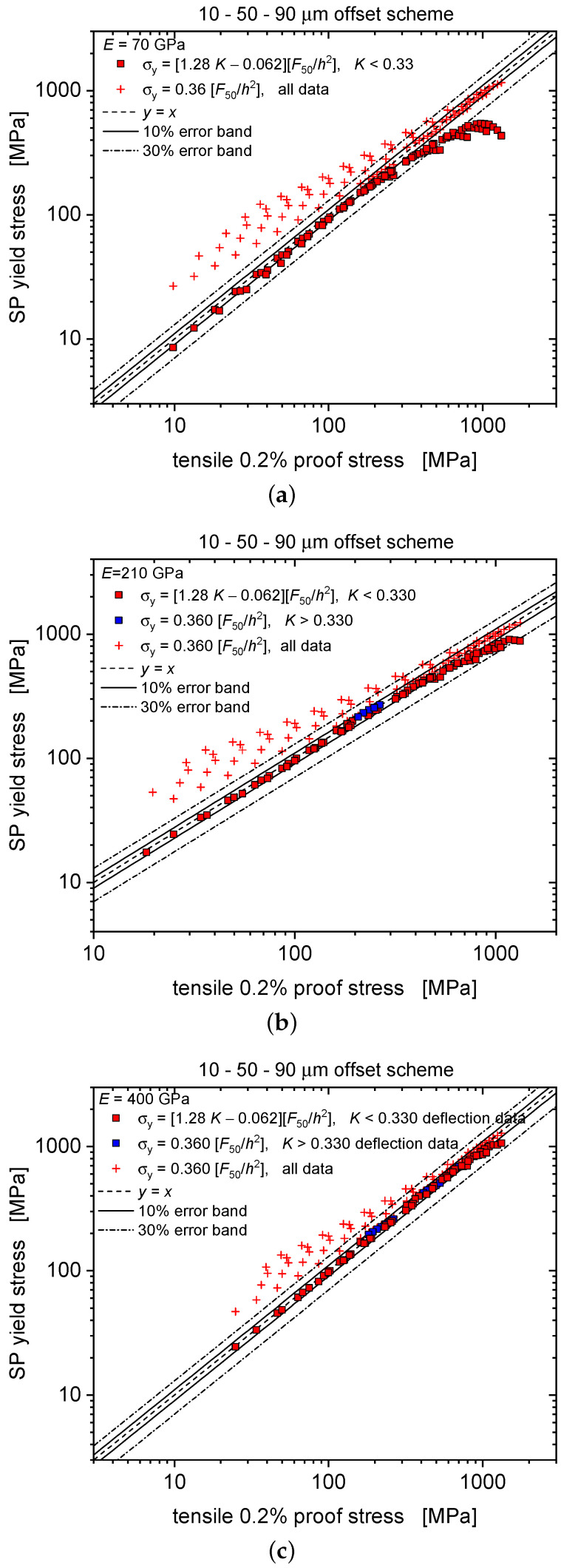
Predictive capability of the displacement-based scheme Equation (3) as applied to deflection data: predicted yield stress vs. numerically implemented proof stress (as determined by Equation (2) for 0.2% of strain) for E=70 GPa (**a**), 210 GPa (**b**) and 400 GPa (**c**), respectively. If compared with the traditional formula (Equation (1)), cf. red cross symbols, the degree of agreement is much superior with the *K* dependent formula.

**Figure 4:**
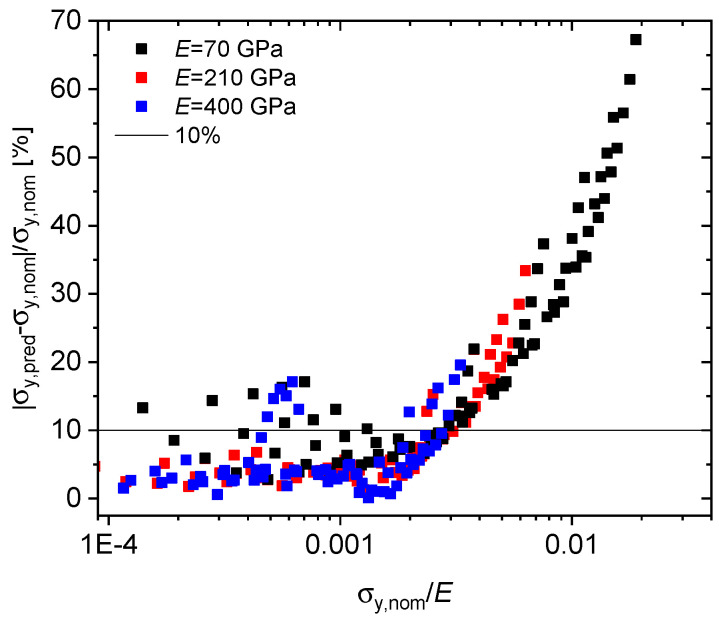
Error percentage of yield stress predicted by the displacement-based formula (Equation (3)), when applied to deflection data. Higher error percentages are noted for $\sigma_{y,\mathrm{nom}}/E>0.003$.

Yield stresses predicted by the displacement-based formula of Equation (3), however, as applied to deflection data, thereby neglecting the difference between displacement and deflection, are compared with the actually implemented tensile proof stresses in Figure 3. Similar to the results based on force–displacement curves presented in Reference [17], the force–deflection curve results (square symbols) exhibit a much improved correlation quality as compared to Equation (1) (red crosses, for which for the sake of comparability α was also set to 0.36). Agreement further improves with increasing elastic modulus. Nevertheless, as seen in Figure 4, error percentages for relative strengths $\sigma_{y,\mathrm{nom}}/E$ in excess of 0.003 may exceed 10% and still rise up to 67% for 0.02, pointing at the need for refining the yield prediction scheme specifically for deflection-based data.

### 4.2. New Formula for Deflection Data

**Figure 5:**
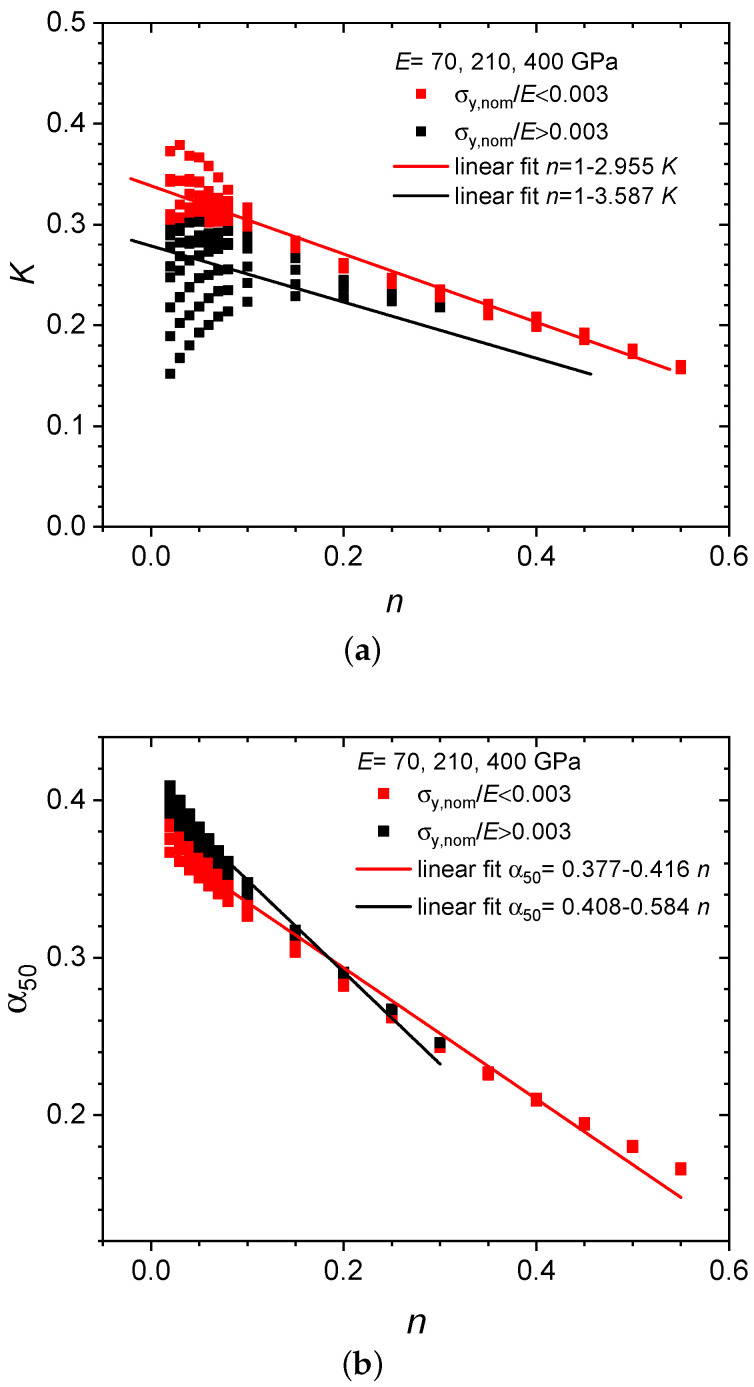
Linear regression fits for *K* vs. *n* (**a**) and $\alpha_{50}$ vs. *n* (**b**).

To develop a formula featuring improved predictive capability for the whole strength and hardening domain, the entire data set, that is, all three Young’s moduli were analyzed together. Linear relations for *K* and the yield coefficient $\alpha_{50}=\sigma_{y}/\left[F_{50}/h^{2}\right]$ as functions of the hardening exponent *n* were obtained (Figure 5) and used to identify again stepwise linear functions for estimating $\sigma_{y}$ in terms of Equations (5)–(7). Contrary to what was found for the displacement-based data [17], it turned out impossible to find a common formulation for all the deflection-based data. We rather have to distinguish a low-strength and a high-strength regime with $\sigma_{y,\mathrm{nom}}/E<0.003$ and $\sigma_{y,\mathrm{nom}}/E>0.003$, respectively.

#### 4.2.1. Low-Strength Regime with $\sigma_{y,\mathrm{nom}}/E<0.003$

While we continue to use the designation $F_{50}$ it is important to note that the characteristic forces are now determined by applying an offset (50 μm) in deflection rather than displacement. For the low-strength regime with $\sigma_{y,\mathrm{nom}}/E<0.003$, the *K* dependence of *n* is approximated by (see Figure 5)

(5) n=1−2.955KforK<0.338.

The *n*-dependence of $\alpha_{50}$ reads

(6) $$\alpha_{50}=0.377-0.416\;n\;\mathrm{for}\;n>0\;.$$

Here, a step at K=0.338 is required by the necessity to consider positive *n* values only. Upon eliminating *n* the *K*-dependence of $\alpha_{50}$ leads to the following yield stress as derived from the force at 50 μm deflection offset:

(7) $$\sigma_{y}=\left\{\begin{array}{cc} \left[1.229K-0.039\right]\frac{F_{50}}{h^{2}} & \mathrm{for}\;K<0.338\;,\\ 0.377\frac{F_{50}}{h^{2}} & \mathrm{otherwise}\;. \end{array}\right.$$

This formulation only applies to low-strength alloys with $\sigma_{y,\mathrm{nom}}/E<0.003$. It closely resembles Equation (3), noting that the parameters are now optimized for offsets applied to force–deflection curves for $\sigma_{y,\mathrm{nom}}/E<0.003$.

As shown in Figure 5, the same procedure has been applied individually to the partial data set of the high-strength regime $\sigma_{y,\mathrm{nom}}/E>0.003$. Accordingly, one obtains n=1−3.587K for K<0.279 and the *K*-dependent yield coefficient $\alpha_{50}=0.408-0.584\;n$ for n>0. The *K*-dependence of $\alpha_{50}$ leads to the following *K*-dependent yield stress as derived from the force at 50 μm deflection offset: $\sigma_{y}=\left[2.095K-0.176\right]F_{50}/h^{2}$ for K<0.279, and $\sigma_{y}=0.408F_{50}/h^{2}$ otherwise. Here a step at K=0.279 again ensures positive *n* values. In the present high-strength case, however, errors in predicting the yield stresses have turned out to remain unacceptably high.

**Figure 6:**
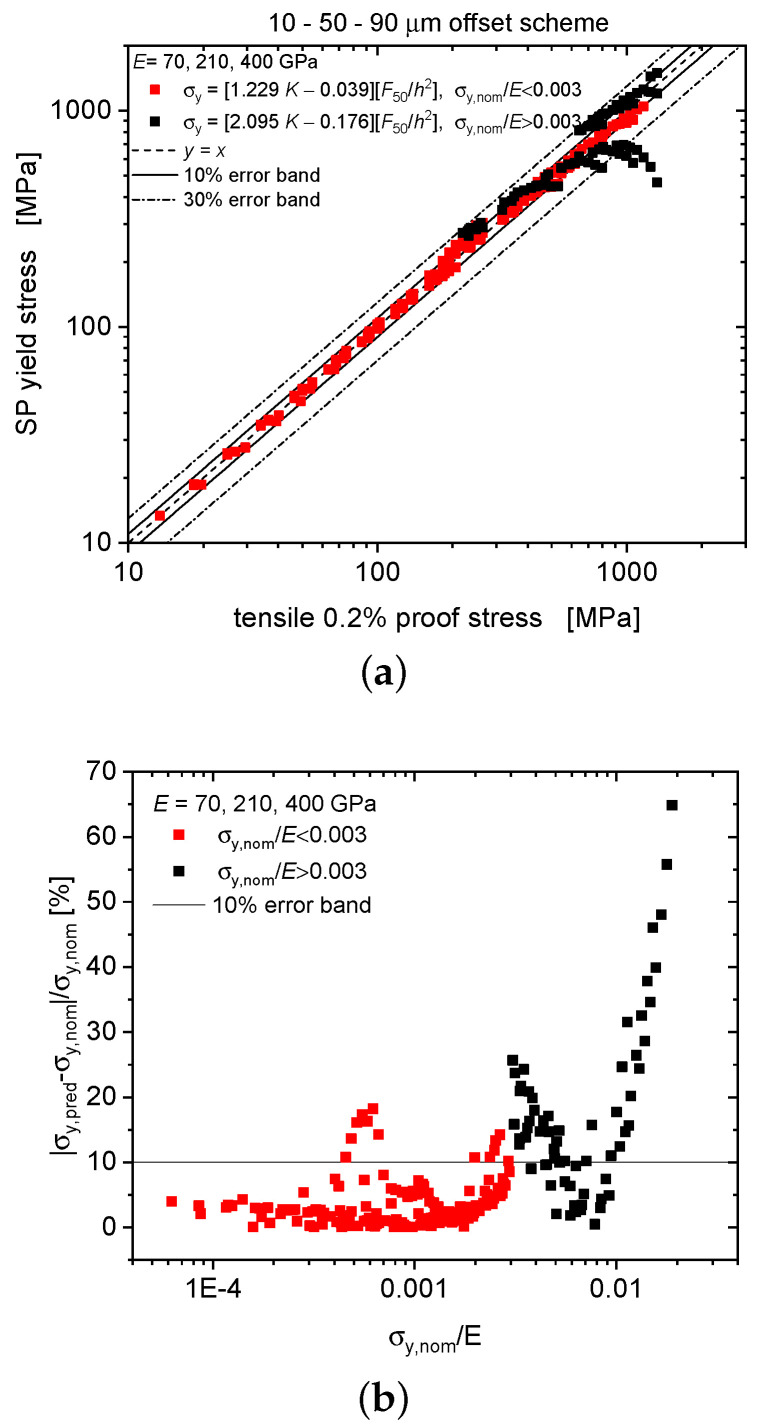
Predictive performance of the deflection-based formula: predicted yield stresses vs. nominal tensile proof stresses (**a**), error percentage of the predicted SP yield stress vs. normalized nominal proof stress $\sigma_{y,\mathrm{nom}}/E$ (**b**).

A comparison of the yield stresses as predicted by the deflection-based formula and the tensile proof stresses implemented in the numerical modelling is presented in Figure 6. A superior prediction capability is visible for the low-strength regime that deteriorates, however, with increasing values of $\sigma_{y,\mathrm{nom}}/E$ and becomes unacceptably poor for the domain $\sigma_{y,\mathrm{nom}}/E>0.003$, reaching up to 65%. A different approach is therefore required for this domain.

#### 4.2.2. High-Strength Regime with $\sigma_{y,\mathrm{nom}}/E>0.003$

In order to decrease the error percentages for the region $\sigma_{y,\mathrm{nom}}/E>0.003$, a slope factor (*S*) as defined in Equation (8) has proven appropriate. It is computed from the previously introduced force levels $F_{10}$ and $F_{50}$ as

(8) $$S=\frac{F_{50}-F_{10}}{F_{50}}\;.$$

**Figure 7:**
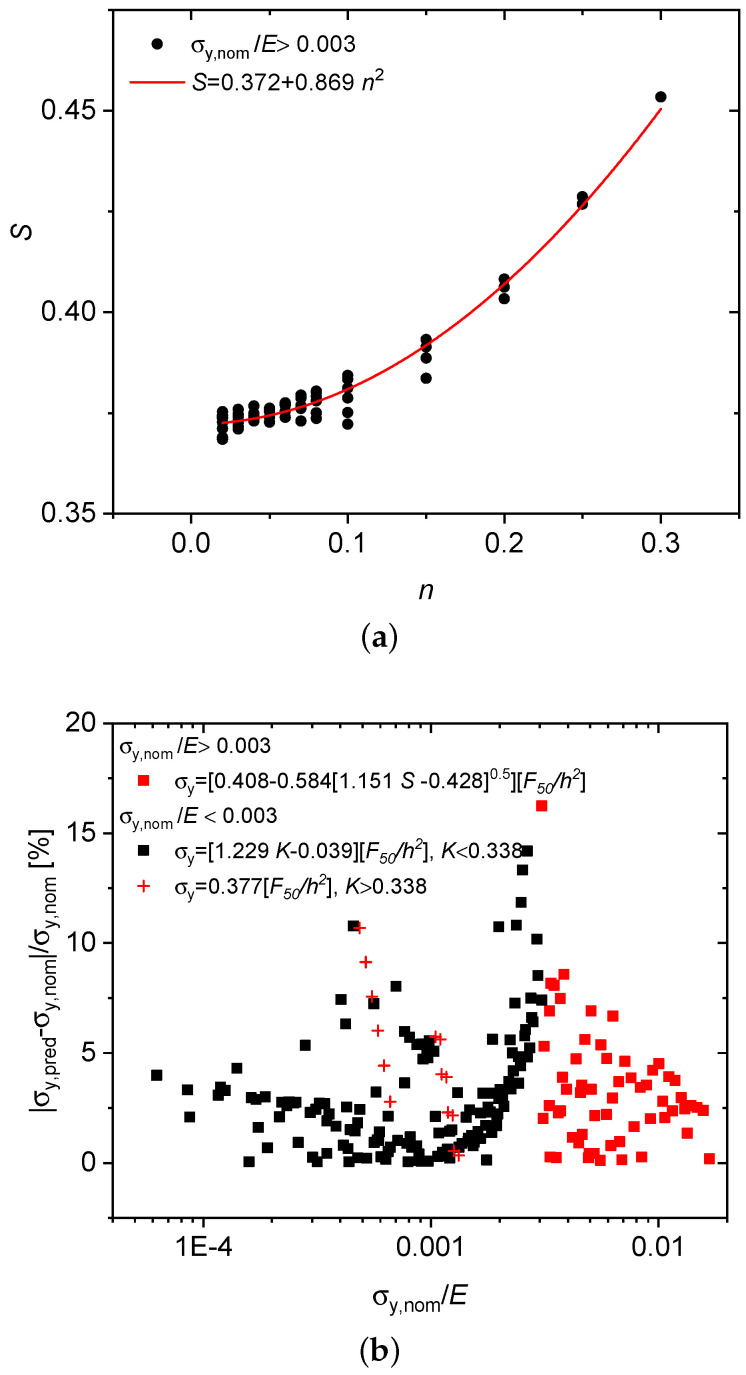
Predictive performance of the slope-dependent formula: hardening exponent *n*-dependence of the slope factor *S* (**a**), error percentage of the predicted SP yield stress vs. normalized proof stress $\sigma_{y,\mathrm{nom}}/E$ (**b**).

With this a parabolic fit can be done to describe its *n* dependence, see Figure 7a

(9) $$S=0.372+0.869\;n^{2}\;.$$

With $\alpha_{50}=0.408-0.584\;n$ for n>0 (cf. Figure 5b) and Equation (9) *n* is then eliminated to obtain $\alpha_{50}$ in terms of the slope factor *S*,

(10) $$\alpha_{50}=0.408-0.584\;{\left[1.151S-0.428\right]}^{1/2}\;,$$

which, by utilizing Equation (10), leads to an *S*-dependent expression for the yield stress in the high-strength regime $\sigma_{y,\mathrm{nom}}/E>0.003$

(11) $$\sigma_{y}=\left\{\begin{array}{cc} \left[0.408-0.584\;{\left[1.151S-0.428\right]}^{1/2}\right]\frac{F_{50}}{h^{2}} & \mathrm{for}\;S>0.372\;,\\ 0.408\frac{F_{50}}{h^{2}} & \mathrm{otherwise}\;. \end{array}\right.$$

In this way it has been possible to effectively reduce the maximum error percentage to 16% down from the former 65% as seen in Figure 7b.

#### 4.2.3. Transition Regime with $0.0025<\sigma_{y,\mathrm{nom}}/E<0.003$

**Figure 8:**
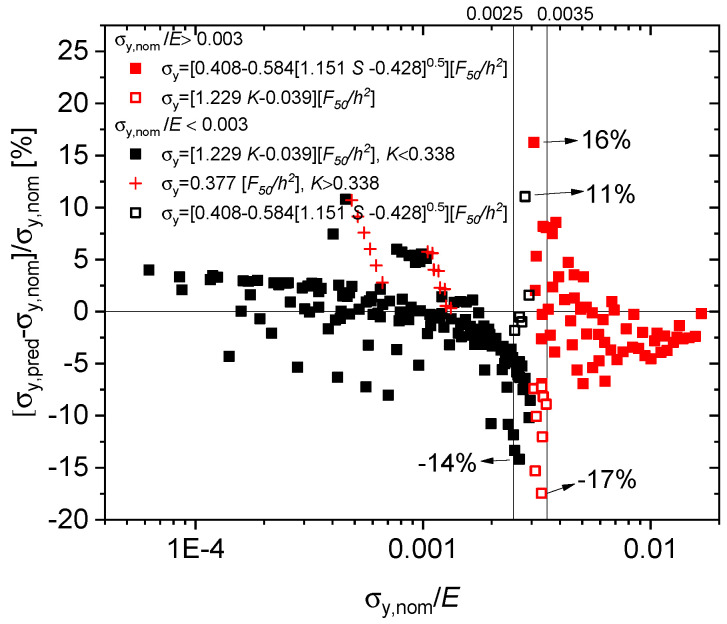
Predictive performance of the curvature- and slope-based formulas in the transition region.

Figure 8 demonstrates the predictive performance of the method wherein a transition region with $0.0025<\sigma_{y,\mathrm{nom}}/E<0.0035$, both the curvature- and the slope-based formulas are used for low- and high-strength alloys. In absolute terms, error percentages increase from 8.6% to 17.5% if the curvature-based formula Equation (7) is applied to high-strength alloys, and similarly from 14.2% to 16.2% if the slope based formula Equation (11) is applied to low-strength alloys. As a consequence, the overall performance of the scheme remains satisfactory even if the elastic properties of the material under test are poorly known. In practice, a self-consistent approach is required: starting from an educated guess of $\sigma_{y,\mathrm{nom}}/E$ to identify the applicable formula, one calculates the resulting $\sigma_{y,\mathrm{nom}}/E$ to verify if the relevant formula has been chosen.

The high-strength alloys with $\sigma_{y,\mathrm{nom}}/E>0.003$ tend to possess a low hardening exponent *n*, with significant *E* dependence of their initial deformation behaviour. Due to their low hardening capacity they exhibit a pronounced non-uniform elastic–plastic transition behaviour during triaxial SP deformation leading to increased uncertainty in predicting their yield stresses. High-strength alloys are better characterized by the initial slope, indicative of their (comparatively weak) hardening behaviour.

The low-strength alloys, however, tend to possess a larger hardening capacity, thus larger *n*. They are dominated by their smoothly proceeding plastic behaviour (less significance of elastic modulus *E*), which altogether is more sensitively characterised by the curvature of the force-deflection curve.

## 5. Conclusions

In this work, a new methodology to predict yield stresses from the force–deflection curves obtained by Small Punch (SP) testing has been presented. This is in contrast to the more common practice, which‘favours force–displacement curves for their simple experimental set-up. As a consequence, however, frame compliance and any insufficiently accurate compensation thereof, introduces significant uncertainties, which adversely affect the usefulness of the SP test for yield stress determination.

To overcome this deficiency, the present approach relies on force–deflection data to determine characteristic force levels from the initial part of the curve to reflect both the slope and the curvature. This is in contrast to the conventional approach, which relies on a single force level, thereby discarding slope and curvature characteristics. The predicted yield stresses for different types of materials, that is, high and low-strength alloys, are found in good agreement with the actual proof stresses with a maximum error of 16%.

In the present numerical analysis and validation of the method, an attempt was made to strike a proper balance between the simplicity of the evaluation scheme formulated and the complexity needed to arrive at an acceptable level of uncertainty. With a view to experimental practice, a 10% uncertainty level has been targeted from the numerical point of view, which is considered satisfactory, if one notes that experimental uncertainties in determining the deflection slope are at least as big (e.g., due to the initial settling of the test piece in the load train), while uncertainties associated with previous evaluation schemes are much larger by a factor of three and more.

In the authors’ previous work [17] on the derivation of yield stresses from displacement data a common formulation was found for the entire strength parameter range, whereas here the deflection sensitively depends on disc thinning and plastic indentation, which require a distinction be made between a high and a low-strength regime. In this context, force–displacement represents a response-stimulus couple, whereas force–deflection data is based on two (different types of) responses of the material to the external stimulus (displacement). The concomitant loss of significance of the deflection data is a source of ambiguity that required us to distinguish the low/high-strength regimes.

In conclusion, the present deflection-based approach leads to give rise to more robust yield stress prediction as compared to the displacement-based approach, because the basic performance of the two predictive schemes is about the same in terms of the established error percentages, whereas experimental practice clearly points at significant uncertainties associated with the correction of the frame compliance. As the deflection measurement is not affected by parasitic compliance, it constitutes the method of choice.
